# Combination of ataxia telangiectasia and Rad3-related inhibition with ablative radiotherapy remodels the tumor microenvironment and enhances immunotherapy response in lung cancer

**DOI:** 10.1007/s00262-024-03864-6

**Published:** 2024-11-02

**Authors:** Jenny Ling-Yu Chen, Chun-Kai Pan, Li-Cheng Lin, Yu-Sen Huang, Tsung-Hsuan Huang, Shu-Jyuan Yang, Sung-Hsin Kuo, Yu-Li Lin

**Affiliations:** 1 Department of Radiology, National Taiwan University College of Medicine, Taipei, Taiwan; 2 National Taiwan University Cancer Center, National Taiwan University College of Medicine, Taipei, Taiwan; 3 Division of Radiation Oncology, Department of Oncology, National Taiwan University Hospital, Taipei, Taiwan; 4 Department of Medical Research, National Taiwan University Hospital, No. 7 Chung-Shan S. Rd., Taipei, 100 Taiwan; 5 Institute of Biomedical Engineering, College of Medicine and College of Engineering, National Taiwan University, Taipei, Taiwan; 6 Department of Medical Imaging, National Taiwan University Hospital, Taipei, Taiwan

**Keywords:** ATR inhibition, Ablative radiotherapy, Immune checkpoint inhibitor, Lung cancer, STING pathway, Tumor microenvironment

## Abstract

We investigated the combined effects of ataxia telangiectasia and Rad3-related (ATR) inhibition, ablative radiotherapy, and immune checkpoint inhibitor (ICI) therapy against lung cancer. ATR inhibitor was administered combined with ablative radiotherapy to assess its radiosensitizing effect on lung cancer cells. Treatment response and survival were evaluated in vivo using A549 xenograft flank tumor and synchronous LLC lung and flank tumor mouse models. Mice received ablative radiotherapy (12 Gy/d for 2 d), ATR inhibitor, and ICI. The tumor microenvironment was assessed in irradiated flank and non-irradiated lung tumors. Programmed death-ligand 1 expression was upregulated after irradiation. ATR inhibition attenuated this upregulation. ATR inhibitor pretreatment decreased cell survival after irradiation by inhibiting DNA double-strand break repair, inducing mitotic cell death, and altering cell cycle progression. ATR inhibition enhanced radiation-induced damage-associated molecular patterns determined by high mobility group box 1 quantification and activated the cyclic GMP-AMP synthase-stimulator of interferon genes pathway. Combined ATR inhibition and ablative radiotherapy inhibited tumor growth and improved survival in mice. Adding ICI therapy further enhanced local antitumor effects, reducing the metastatic lung tumor burden and remodeling the tumor microenvironment through immunogenic cell death induction and enhanced immune cell infiltration. Triple therapy increased immune cell infiltration in distant non-irradiated lung tumors and stimulated the generation of protective T-cell immunity in splenocytes. Safety analysis showed minimal toxicity. ATR inhibition enhanced the efficacy of ablative radiotherapy and immunotherapy in lung cancer. These findings underscore the importance of combination therapies for enhancing systemic antitumor immune responses and outcomes.

**Supplementary Information:**

The online version contains supplementary material available at 10.1007/s00262-024-03864-6.

## Introduction

Lung cancer is the most common type of cancer and a major cause of cancer-related deaths. Approximately 30–40% of patients exhibit metastasis at diagnosis, with most lesions being unresectable [1, 2]. Advances in non-surgical techniques and targeted therapies have improved survival. Immune checkpoint inhibitors (ICIs) are promising; however, primary or acquired resistance limits their efficacy [3]. Combining ICIs with radiotherapy or targeted therapies can overcome ICI resistance, improving treatment responses [4, 5].

Ablative radiotherapy inhibits local tumor growth and enhances systemic antitumor immune responses [6]. These effects are mediated via immune stimulation by radiation-induced proinflammatory factor production, damage-associated molecular patterns (DAMPs) [7, 8], and greater exposure to tumor-derived antigens (released upon radiotherapy-induced immunogenic cell death).

Mammalian cells respond to DNA damage through complex mechanisms. Ataxia telangiectasia and Rad3-related (ATR) is critical for repairing radiation-induced DNA double-strand breaks [9, 10]. ATR inhibitors (ATRi) enhance antitumor immunity in various models [5, 11–15]. Preclinical studies demonstrated enhanced antitumor efficacy of combined ATR inhibition and ablative radiotherapy without an increase in toxicity [16–18]. However, the effect of ATRi-mediated radiosensitization on the tumor microenvironment remains elusive. Herein, we aimed to investigate the combined effects of ATR inhibition and ablative radiotherapy, in addition to exploring the efficacy of combined ATR inhibition, ablative radiotherapy, and ICI in lung cancer. We hypothesized that combined ATR inhibitors would enhance ablative radiotherapy-induced tumor cell death and potentiate the tumor microenvironment, and the addition of ICI therapy would strengthen systemic antitumor immunity.

## Materials and methods

### Cell culture

Non-small cell lung cancer cells including A549 human adenocarcinoma, H520 human squamous cell carcinoma, and LLC murine lung carcinoma cells were purchased from the food industry research and development institute (Taiwan) and maintained in Dulbecco’s modified Eagle’s medium supplemented with 10% heat-inactivated fetal bovine serum (FBS) and penicillin/streptomycin at 37 °C in a humidified atmosphere containing 95% air and 5% CO_2_.

### Cell viability

Cells (2 × 10^3^) were seeded in 96-well plates and treated with different concentrations of ATR inhibitor berzosertib (MedChemExpress, USA). After 48 h of treatment, 10 μL of the Cell Counting Kit-8 (Sigma-Aldrich, USA) was added, followed by incubation for 2 h. Optical density was measured at 450 nm. IC_50_ values were calculated using the least square fit of four-parameter sigmoidal curves plotted using GraphPad Prism 5 (GraphPad Software, USA).

### Colony formation

Based on the previous studies demonstrating radiation sensitization, berzosertib doses of 40 and 80 nM were selected [16, 18]. After 16 h berzosertib treatment, cells were seeded in 6-well plates (200–1000 cells/well) and irradiated with 0, 2, 4, 6, or 8 Gy of *γ*-rays at a rate of 210 cGy/min using a 137 Cs Gammacell-40 irradiator (CIS Bio International, France). After 10–14 d, colonies containing > 50 cells were fixed and stained with glutaraldehyde and crystal violet (Sigma-Aldrich, USA), then analyzed using an ImmunoSpot S6 UV Reader (Cellular Technology Ltd., USA).

### Flow cytometry

To determine cell surface PD-L1 expression, ATR inhibitor berzosertib (80 nM)- or radiotherapy (6 Gy)-treated cells were incubated with APC-conjugated anti-PD-L1 antibodies (BD Biosciences, USA) at room temperature (20–22 °C). After washing and centrifugation, the cells were analyzed via fluorescence-activated cell sorting using the BD FACSLyric Clinical Flow Cytometry System (BD Biosciences). Data were analyzed using FlowJo v10 (Tree Star, USA).

### Micronuclei analysis

The presence of fragmented micronuclei was used as the criterion for cells undergoing mitotic catastrophe. After 16 h of berzosertib treatment, cells were irradiated with 6 Gy, harvested 8 h post-irradiation, and stained with bisBenzimide Hoechst 33,342 trihydrochloride (1:2000 dilution, DNA content marker, Sigma-Aldrich, USA) and Alexa Fluor 488-conjugated anti-phospho-histone *γ*H2AX Ser139 (1:500 dilution, DNA double-strand break marker, Sigma-Aldrich) at room temperature in the dark. Images were acquired using the ImageXpress nano-automated imaging system (molecular devices, USA) and analyzed automatically using MetaXpress software (molecular devices). The number of *γ*-H2AX foci per cell was determined using the granularity module. Micronuclei were identified and analyzed using the micronuclei module, based on size, intensity, and distance from the main nuclei. Micronuclei sizes ranged between 1/16th and 1/3rd of the mean diameter of the main nuclei. They exhibited equal or greater staining intensity compared to the main nuclei and were not connected to the main nuclei, with clearly distinguishable boundaries [19].

### Western blotting

Cell lysates containing 100 μg of protein were separated via sodium dodecyl sulfate polyacrylamide gel electrophoresis on a 10–12% polyacrylamide gel, transferred onto polyvinylidene fluoride membranes, and immunoblotted with antibodies against ATR/p-ATR (Invitrogen, USA), HMGB1, cGAS, STING, p-STING, TBK1, p-TBK1, IRF3, and pIRF3 (Cell Signaling Technology, USA). Bound antibodies were detected via enhanced chemiluminescence using a peroxidase-coupled secondary antibody (Boehringer, Germany). GAPDH (cell signaling technology) was used as a loading control.

### Cell cycle analysis

Cells (10^6^/mL) were irradiated with 6 Gy either alone or after berzosertib pretreatment for 16 h. Cells were harvested 24 h post-irradiation, fixed in 70% ethanol, and stained with propidium iodide (1:50 dilution, nucleic acids staining marker, Sigma-Aldrich). Cell cycle distribution was determined using flow cytometry to analyze DNA content on the BD FACSLyric clinical flow cytometry system (BD Biosciences). Data analysis was conducted using the cell cycle platform in FlowJo v10 software (tree star), which provided cell fractions in subG1, G0/G1, S, and G2/M phases.

### Animal experiments

All animal experiments were approved by our Institutional Animal Care and Use Committee (approval no. 20230216). Female C57BL/6 and nude mice (6 weeks old) were purchased from the National Laboratory Animal Center (Taiwan) and housed under specific pathogen-free conditions in an Association for Assessment and Accreditation of Laboratory Animal Care-approved facility. Mice were group-housed (five per cage) under a 12 h light/dark cycle (lights on at 08:00 am) and provided ad libitum access to sterilized chow (LabDiet 5001; LabDiet, USA) and water.

For the A549 flank tumor model, nude mice were subcutaneously inoculated with 2 × 10^6^ A549 cells (passage 3) in the hind flank. A solid tumor (36 ± 16 mm^3^) developed 14 d after inoculation. Tumor-bearing mice were randomized into four groups: control, ATRi (20 mg/kg/d berzosertib for 5 d by oral gavage), ablative radiotherapy (12 Gy/d for 2 d), and ATRi plus ablative radiotherapy. The berzosertib dose administered in vivo by oral gavage ranged from 20 to 60 mg/kg/day for 3–10 days when combined with chemotherapeutics or radiotherapy [13, 16, 20]. The dose of 20 mg/kg/d for 5 d was selected to optimize radiosensitizing and immune-modulatory effects while minimizing systemic toxicities.

For the synchronous LLC lung and flank tumor model, C57BL/6 mice first received an intrapulmonary injection of 4 × 10^3^ LLC cells for tumor development. Two days later, mice were subcutaneously injected with 4 × 10^5^ LLC cells (passage 3) in the flank. Solid tumors (35 ± 13 mm^3^) developed 10 d after inoculation. Tumor-bearing mice were randomized into six groups: control, ATRi (20 mg/kg/d berzosertib for 5 d by oral gavage), ATRi plus anti-PD-L1 (200 μg administered intravenously 3 d apart for a total of 2 injections), ablative radiotherapy (12 Gy/d for 2 d), ATRi plus ablative radiotherapy, and ATRi plus anti-PD-L1 and ablative radiotherapy. The monoclonal antibodies against mouse PD-L1 (clone 10F.9G2, isotype rat IgG2b kappa) were purchased from Bio-Xcell (USA) [4, 21]. PD-L1 is the ligand for PD-1, an inhibitory receptor expressed on activated T cells. Inhibition of the PD-L1/PD-1 pathway enhances T-cell activation, cytokine production, and cytolysis, which has been leveraged for cancer immunotherapies [22]. The anti-PD-L1 dose of 200 μg was selected based on the previous studies that investigated the combined effects of ICI, radiotherapy, and ATR inhibition [23].

Radiation was administered using a 137 Cs Gammacell-40 irradiator (CIS Bio International) [4, 8]. Mice were anesthetized via intramuscular injection of a cocktail of tiletamine/zolazepam (7.5 mg/kg Zoletil; Virbac, France) and xylazine (0.12 mg/kg Rompun; Bayer, Germany). A custom-made lead shield with the size of a non-shielded field of 1.0 cm × 0.5 cm was placed to deliver the ablative RT to the hind tumor while minimizing radiation dose to other body areas. Consistency in radiation shielding and delivery was maintained across all mice receiving radiotherapy. The dose rate was 210 cGy/min, calibrated and validated using the thermoluminescent dosimeter TLD 100H (Thermo Fisher Scientific, Waltham, MA, USA), and extended dose range films (CareStream, Rochester, MA, USA), as per the manufacturer’s protocol and published studies [24, 25]. Mice received 12 Gy per fraction on 2 consecutive days for a total of 24 Gy. The total irradiation time was 5.7 min per fraction.

Tumor volume and body weight were measured every 2–3 d. Humane endpoints were determined according to a clinical scoring system based on our Institutional Animal Care and Use Committee protocol. Mice with a weight loss of ≥ 20%, tumor diameter of > 20 mm, tumor severely impairing ambulation, or severe ulceration were euthanized.

### Immunophenotyping

To isolate tumor-infiltrating lymphocytes (TILs), solid lung and flank tumors were excised 8 days post-irradiation. Single-cell suspensions were prepared using a gentle MACS dissociator and a murine tumor dissociation kit (Miltenyi Biotec, Germany), followed by density gradient centrifugation on an 80%/40% Percoll (GE Healthcare, USA) gradient. To isolate splenocytes, the spleen was removed 8 days post-irradiation. Single-cell suspensions were prepared by forcing the spleen through a 400 μm stainless-steel mesh strainer. Erythrocytes were lysed with a hypotonic buffered solution. The lymphocytes were washed with Hank’s balanced salt solution and resuspended in a medium supplemented with 10% FBS. Cells were stained with fluorescently labeled antibodies (BD Biosciences) and evaluated for the expression of CD3/4/8/11b/11c/19/44/45/49/62L and F4/80 via multiparameter flow cytometry using the BD FACSLyric clinical flow cytometry system (BD Biosciences). Data were analyzed using FlowJo v10 (tree star). The gating strategy is illustrated in Supplementary Fig. S1. The unstained samples are depicted as negative populations in Supplementary Fig. S2.

### Immunohistochemical staining

Mice bearing LLC tumors at the flank were euthanized 8 days after irradiation. Tumors were fixed in 10% neutral buffered formalin for histopathological examination. To assess the role of the cGAS-STING signaling pathway in the effects of ATRi combined with ablative RT, cGAS expression in LLC flank tumor tissues was examined by immunohistochemical staining using specific antibodies against cGAS (1:100 dilution, ABclonal Technology, USA).

### Biochemical and hematological analyses

Blood was collected via the submandibular vein in mice. Complete and differential blood counts were evaluated 8 days after irradiation using a hematology analyzer (Exigo H400; Boule Medical, Sweden). The following blood parameters were recorded: white blood cell count, platelet count, hemoglobin, and hematocrit. Creatinine, blood urea nitrogen, alanine aminotransferase, and aspartate aminotransferase levels were measured using a dry chemistry analyzer (DRI-CHEM NX-500; Fujifilm, Japan).

### Statistical analysis

All experiments were repeated at least twice. Data are expressed as the means and their standard errors. Between-group comparisons were performed using the Mann–Whitney *U*-test. Survival curves were plotted using the Kaplan–Meier method and compared using the log-rank (Mantel-Cox) test. All statistical analyses were conducted using SPSS for Windows (version 17.0; SPSS Inc., USA). *P* ≤ 0.05 was considered statistically significant.

## Results

### ATR inhibitor sensitized lung cancer cells to irradiation and attenuated radiation-induced PD-L1 upregulation

**Fig. 1:**
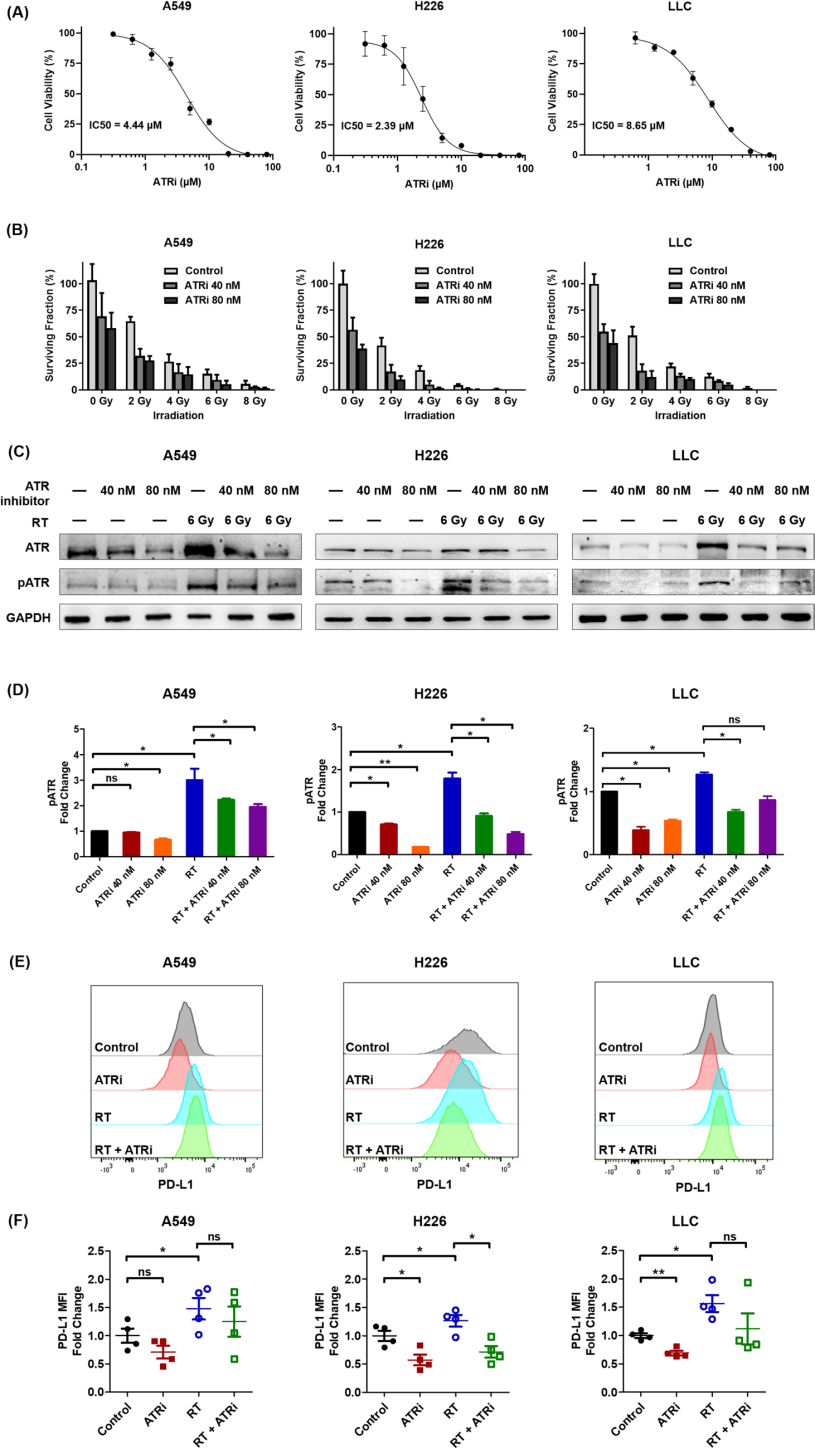
ATR inhibition-induced sensitization to radiotherapy and upregulation of cell surface PD-L1 expression. **A** Cell viability was determined via Cell Counting Kit-8 assay. **B** Response of lung cancer cells to radiation (0, 2, 4, 6, or 8 Gy) in the presence of ATR inhibitor berzosertib (40/80 nM) determined via colony formation assay. **C** ATR/p-ATR (Thr1989) in ATR inhibitor- and radiotherapy-treated cells detected via Western blotting. **D** Relative fold change in p-ATR (Thr1989) protein expression in A549, H226, and LLC cells. **E**, **F** Cell surface PD-L1 expression was analyzed via flow cytometry. Experimental groups contained four samples per group. Data are expressed as the mean ± standard error of the mean. Statistical significance was assessed using the Mann–Whitney *U*-test. *P < 0.05; **P < 0.01; ns, not significant

Cell viability assays showed IC_50_ values of 4.44, 2.39, and 8.65 µM for A549, H226, and LLC cells, respectively (Fig. 1A). Pretreatment with ATR inhibitor before irradiation reduced the surviving fraction of lung cancer cells (Fig. 1B). While irradiation alone markedly enhanced the expression of p-ATR in human and mouse lung cancer cells, ATR inhibition suppressed ATR phosphorylation levels in both non-irradiated and irradiated cells (Fig. 1C, D). PD-L1 expression was upregulated after irradiation; however, this effect was attenuated by ATR inhibitor pretreatment (Fig. 1E, F).

### ATRi-induced radiosensitization was mediated via inhibition of DNA double-strand break repair and mitotic catastrophe

**Fig. 2:**
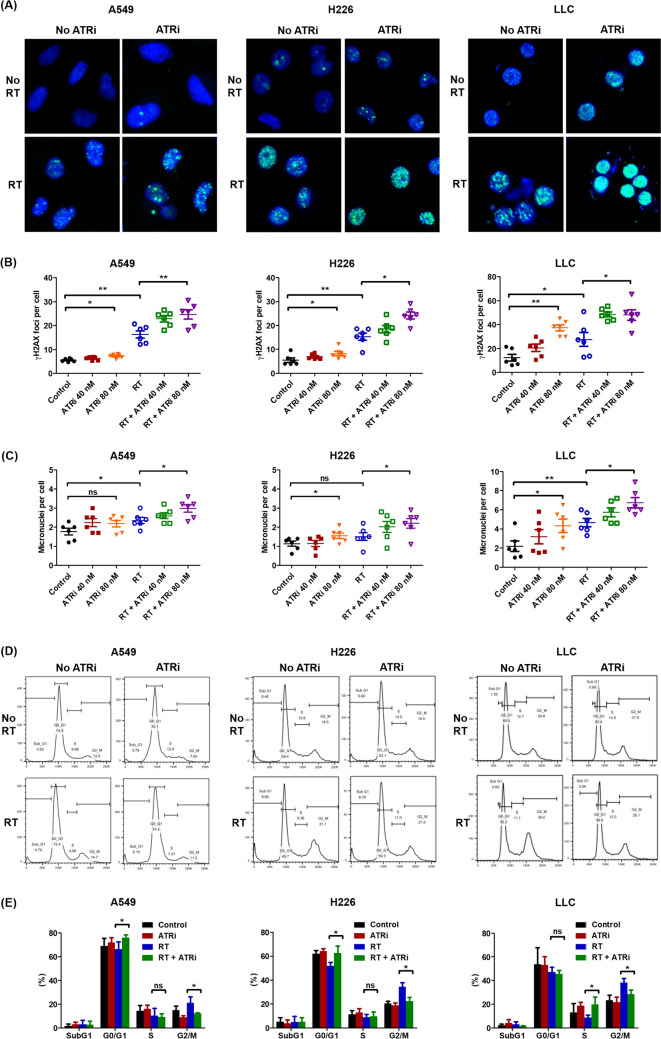
Micronuclei, *γ*H2AX foci, and cell cycle analysis after combined ATR inhibition and ablative radiotherapy. **A** Representative immunofluorescence merged images of *γ*-H2AX foci shown in green (AlexaFluor488) and nuclei in blue (Hoechst 33,342) in lung cancer cells treated with ATR inhibitor and radiotherapy, alone and in combination. **B** Quantification of *γ*H2AX foci as a marker of DNA double-strand break after exposure to ionizing radiation. **C** Quantification of abnormal fragmented micronuclei as a marker of mitotic catastrophe. **D**, **E** Representative cell cycle distribution of lung cancer cells treated with ATR inhibitor and radiation, alone and in combination, analyzed via flow cytometry. Experimental groups comprised six samples per group. Data are expressed as the mean ± standard error of the mean. Statistical significance was assessed using the Mann–Whitney *U*-test. *P < 0.05; **P < 0.01; ns, not significant

ATR inhibition before irradiation increased the number of *γ*H2AX foci compared to those after irradiation alone (*P* < 0.01, *P* < 0.05, and *P* < 0.05 for A549, H226, and LLC cells, respectively; Fig. 2A, B). Mitotic cell death analysis showed an increase in the number of cells undergoing mitotic catastrophe after combined treatment (*P* < 0.05 for H226 and LLC cells; Fig. 2C), suggesting that ATRi-induced radiosensitization was mediated via inhibition of DNA double-strand break repair and subsequent mitotic catastrophe. Flow cytometry showed that radiation-induced ATR signaling altered cell cycles, leading to cell cycle arrest in the G2/M phase. ATR inhibitor pretreatment reduced the number of cells in the G2/M phase, driving cells into the G0/G1 phase (*P* < 0.05, A549 and H226 cells) or the *S* phase (*P* < 0.05, LLC cells, Fig. 2D, E).

### ATR inhibition enhanced the antitumor efficacy of combined ICI therapy and ablative radiotherapy, prolonging survival

**Fig. 3:**
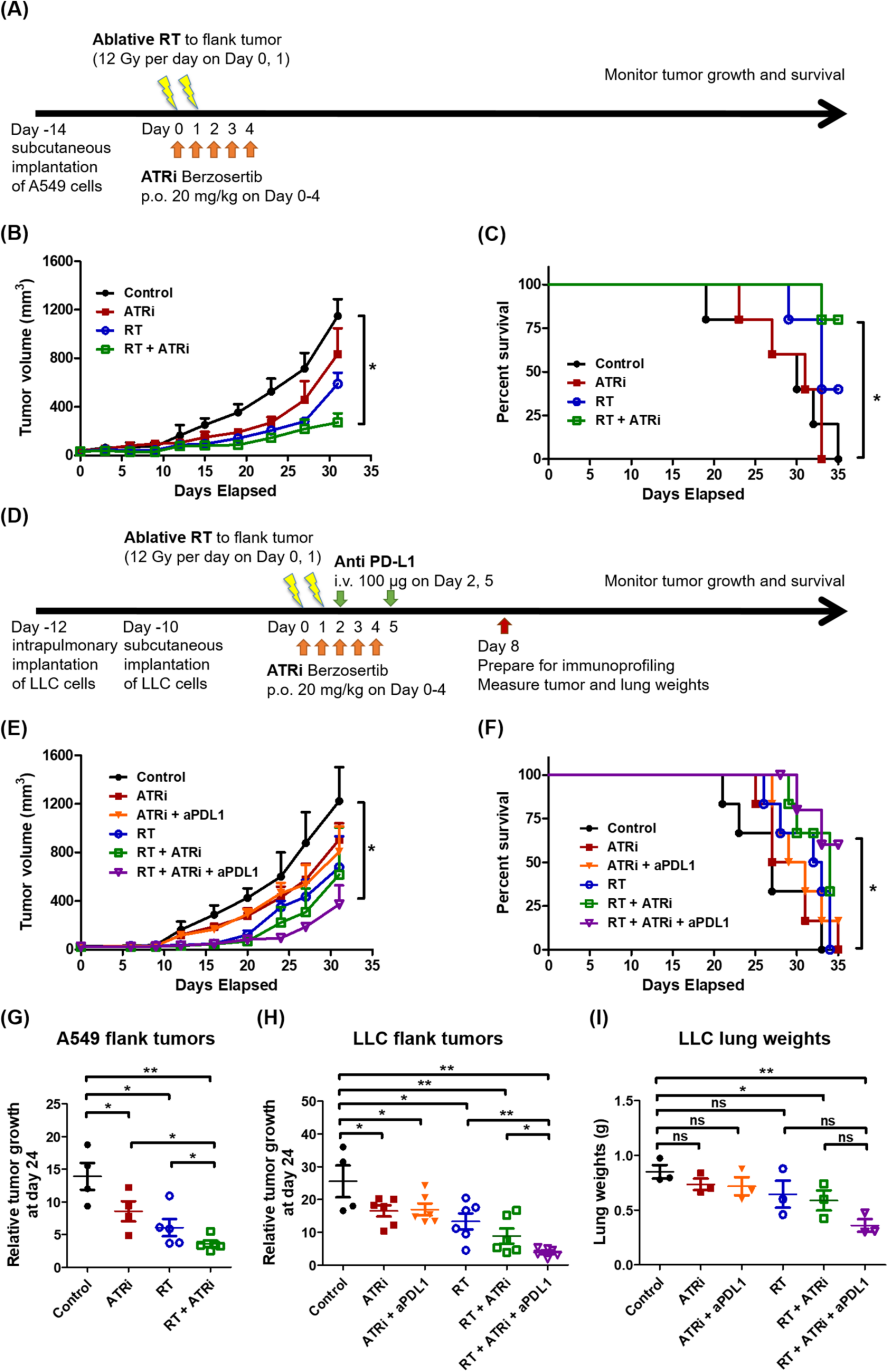
Antitumor effect of ATR inhibition and ICI therapy combined with ablative radiotherapy. **A** Establishment of the subcutaneous A549 xenograft flank tumor mouse model. The experimental groups comprised five mice per group. **B** Tumor growth curves and **C** Kaplan–Meier overall survival curves of subcutaneous A549 xenograft flank tumor model mice. **D** Establishment of the synchronous LLC lung and flank tumor mouse model. The experimental groups comprised six mice per group. **E** Tumor growth curves and **F** Kaplan–Meier overall survival curves of synchronous LLC lung and flank tumor model mice. Relative growth of **G** A549 irradiated flank tumors and **H** LLC irradiated flank tumors on day 24, calculated using the tumor volume on day 24 divided by the initial tumor volume in each individual. **I** Eight days after ablative RT, the lungs were weighed. Lung weight was considered as a proxy of metastatic tumor burden among mice. The experimental groups comprised three mice per group. Data are expressed as the mean ± standard error of the mean. Statistical significance was assessed using the Mann–Whitney *U*-test. *P < 0.05; **P < 0.01; ***P < 0.001; ns, not significant

We employed a subcutaneous A549 xenograft flank tumor mouse model to determine whether ATR inhibition enhanced tumor cell killing by ablative radiotherapy and overall survival (Fig. 3A). Combined ATR inhibition and ablative radiotherapy delayed the growth of A549 tumors (*P* < 0.05; Fig. 3B) and increased overall survival (*P* < 0.05; Fig. 3C), compared to those observed in other groups.

To assess whether ATR inhibition enhanced the systemic antitumor effect of combined ICI therapy and ablative radiotherapy, we used a synchronous LLC lung and flank tumor mouse model (Fig. 3D). Mice implanted with subcutaneous LLC lung and flank tumors were randomized into six groups: control, ATRi, ATRi plus anti-PD-L1 antibody, ablative radiotherapy, ATRi plus ablative radiotherapy, and ATRi plus anti-PD-L1 antibody and ablative radiotherapy. Triple therapy reduced flank tumor growth and prolonged survival relative to other treatments (*P* < 0.05; Fig. 3E, F). On day 24 post-irradiation, ablative radiotherapy eliminated the flank tumor in both A549 and LLC flank tumor models. The addition of ATRi enhanced the local tumoricidal effect of ablative radiotherapy in the A549 flank tumor model (*P* < 0.05; Fig. 3G). Adding anti-PD-L1 to the combination further enhanced local tumor killing in the LLC flank model (*P* < 0.05; Fig. 3H). Lung weight was used as a proxy for metastatic tumor burden. While ATR inhibition or ablative radiotherapy alone had a limited effect on lung tumor progression, the combination of ATR inhibition and ablative radiotherapy significantly reduced the metastatic tumor burden in non-irradiated lung regions (*P* < 0.05) than that in the control group. Moreover, the triple therapy comprising ablative radiotherapy, ATR inhibition, and immune checkpoint inhibitor therapy further reduced the metastatic tumor burden (*P* < 0.01; Fig. 3I), indicating enhanced antitumor immunity and a potential abscopal effect.

### Triple therapy enhanced local immune cell infiltration

**Fig. 4:**
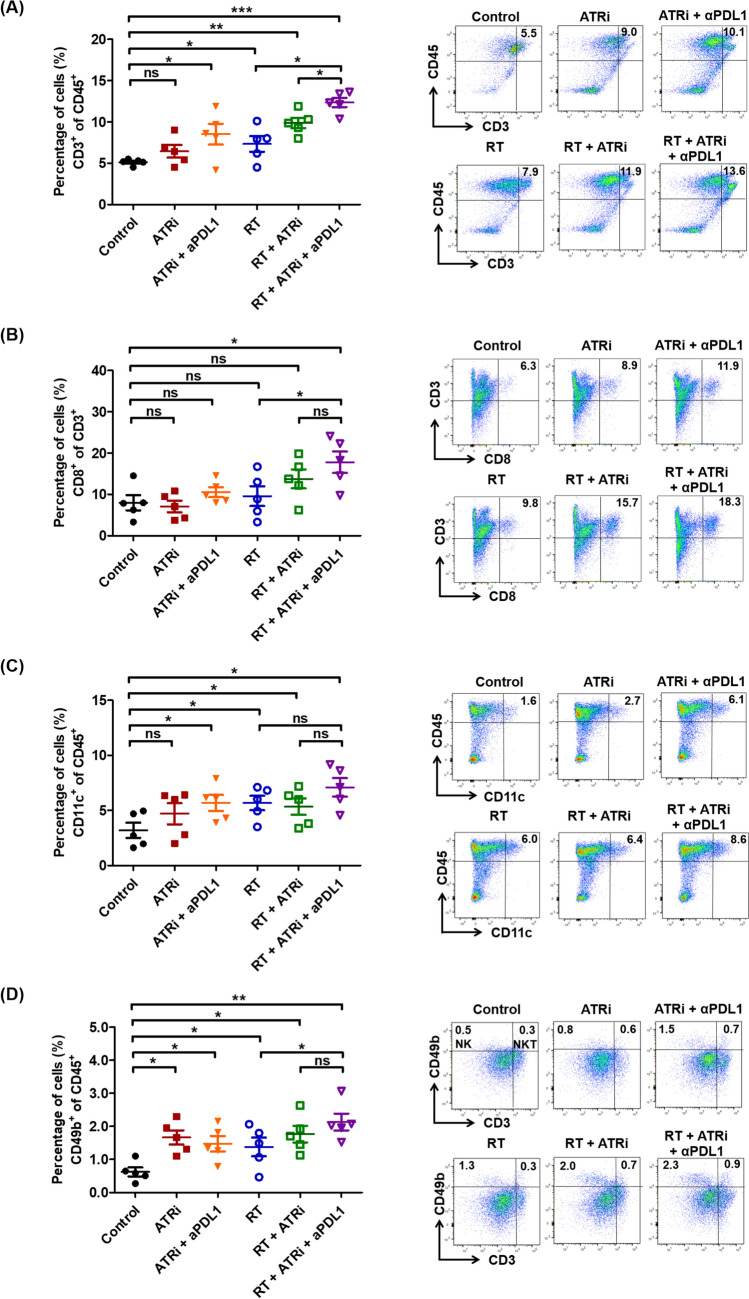
Quantitative flow cytometric analysis of tumor-infiltrating immune cell subpopulations in the flank tumor microenvironment. **A** CD3^+^ T cells, **B** CD8^+^ T cells, **C** CD11c^+^ dendritic cells, and **D** CD3^−^CD49b^+^ NK cells and CD3^+^CD49b^+^ NK T cells. The experimental groups comprised five mice per group. Data are expressed as the mean ± standard error of the mean. *P < 0.05; **P < 0.01; ***P < 0.001; ns, not significant

The migration of immune effector cells into tumor tissues is crucial for potentiating the radiation-induced tumor microenvironment. On day 8 post-irradiation, ATR inhibition or radiotherapy alone had minimal effect on intratumoral immune effector cell recruitment, and the proportions of CD3^+^ (Fig. 4A) and CD8^+^ (Fig. 4B) remained unchanged than those in controls. Combined ATR inhibition and ablative radiotherapy or ICI therapy mildly increased CD3^+^ and CD8^+^ T-cell proportions, with triple therapy further enhancing effector TIL recruitment, achieving the highest CD3^+^ and CD8^+^ cell proportions. An increase in antigen-presenting dendritic cells (CD11c^+^) was noted among tumor-infiltrating dendritic cells (DC) after treatments (Fig. 4C). While notable, the effect was not statistically significant between the control and ATR monotherapy group but was significant in the triple therapy group. We also quantified the natural killer population (CD49b^+^) within the tumor microenvironment, observing a significant increase in total natural killer populations after treatments (Fig. 4D), which was most pronounced after triple therapy.

### ATR inhibition potentiated radiation-induced cyclic GMP-AMP synthase (cGAS)-stimulator of interferon genes (STING) activation

**Fig. 5:**
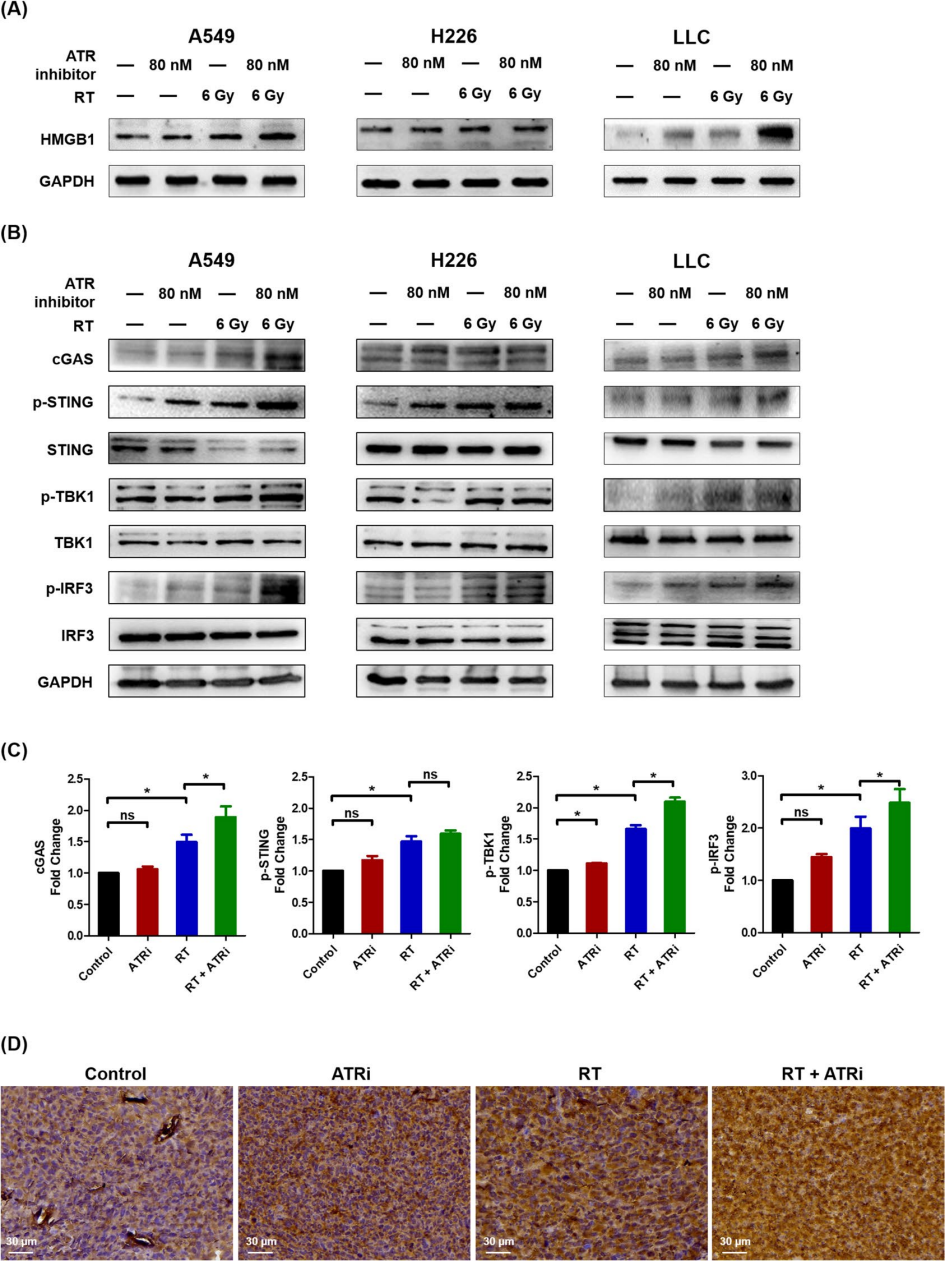
ATR inhibition potentiated radiation-induced cGAS-STING activation. Quantitative analysis of **A** HMGB1 and **B** cGAS, p-STING, STING, p-TBK1, TBK1, p-IRF3, and IRF3 protein expression levels in A549, H226, and LLC cells via Western blotting. **C** Relative fold change in cGAS, p-STING, p-TBK1, and p-IRF3 protein expression in LLC cells. HMGB1, high mobility group box 1; cGAS, cyclic GMP-AMP synthase; STING, stimulator of interferon genes; TBK1, TANK-binding kinase 1; and IRF3, interferon regulatory factor 3. Experimental groups comprised three samples per group. Data are expressed as the mean ± standard error of the mean. *P < 0.05; ns, not significant. **D** Representative immunohistochemical images of cGAS expression in LLC flank tumor tissue treated with ATRi, ablative RT, or both. Scale bars, 30 µm

During ablative radiotherapy, the release of high mobility group box 1 (HMGB1) by dying cells plays a significant role in immunogenic cell death. In lung cancer cells, HMGB1 levels were increased with combined ATR inhibition and ablative radiotherapy compared to radiotherapy alone (Fig. 5A), suggesting a potential synergistic effect between ATR inhibition and radiotherapy in boosting HMGB1-mediated immunogenicity. Furthermore, cell cycle-specific DNA damage and micronuclei formation trigger the cGAS-STING pathway, leading to the phosphorylation of TANK-binding kinase 1 (TBK1) and interferon regulatory factor 3 (IRF3). Combined ATR inhibition and ablative radiotherapy upregulated cGAS, p-STING, p-TBK1, and p-IRF-3, indicating cGAS-STING–p-TBK1–p-IRF3 axis activation in A549, H226, and LLC cells (Fig. 5B, C). In LLC flank tumor model, the expressions of cGAS in the flank tumor tissue treated with both ATRi and ablative RT were significantly increased, compared to the control and monotherapy groups (ATRi or ablative radiotherapy), as shown in Fig. 5D. These in vitro and in vivo findings suggest that ATR inhibition enhanced the radiation-induced DAMPs and activated the interferon signaling to trigger the cGAS-STING–p-TBK1–p-IRF3 pathway, thereby stimulating antitumor immunity.

### Immune activation in distant non-irradiated lung tumors following ATR inhibition, ICI therapy, and ablative radiotherapy

**Fig. 6:**
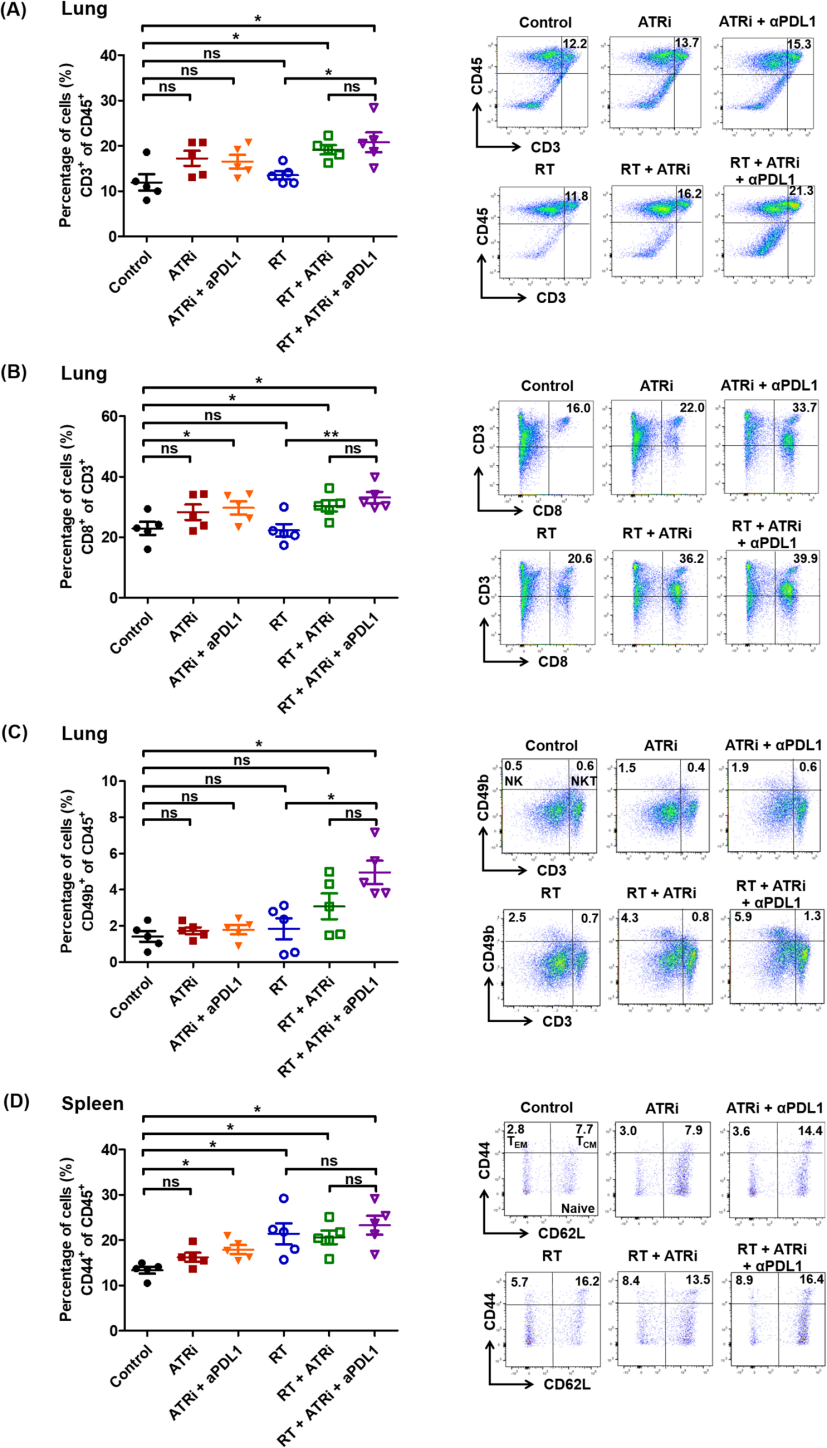
Quantitative flow cytometric analysis of tumor-infiltrating immune subpopulations in the non-irradiated lung tumor microenvironment: **A** CD3^+^, **B** CD8^+^, and **C** CD3^−^CD49b^+^ NK cells and CD3^+^CD49b^+^ NK T cells. **D** Flow cytometric analysis of memory markers on CD8^+^ T cells isolated from the spleen microenvironment. CD44^+^CD62L^–^ and CD44^+^CD62L^+^ cells were classified as effector memory and central memory T cells, respectively. Data are expressed as the mean ± standard error of the mean. The experimental groups comprised five mice per group. Statistical significance was assessed using the Mann–Whitney *U*-test. *P < 0.05; **P < 0.01; ns, not significant

To investigate whether the adaptive antitumor immunity potentiated by combined ATR inhibition and ablative radiotherapy enhanced systemic immunotherapy responses in vivo, we established a synchronous LLC lung and flank tumor mouse model (Fig. 3D). On day 8 post-irradiation, flow cytometry and immunofluorescence staining showed that the control and monotherapy groups (ATRi or ablative radiotherapy) had limited TIL infiltration in the non-irradiated lung tumor microenvironment. Meanwhile, the ATRi plus ablative radiotherapy group showed increased TILs, with higher proportions of CD3^+^ (Fig. 6A) and CD8^+^ (Fig. 6B) cells. ICI addition further enhanced TIL infiltration, achieving the highest CD3^+^ and CD8^+^ cell proportions. Enhanced infiltration was also noted with the CD49b^+^ natural killer population (Fig. 6C) in the triple therapy group.

### Evaluation of memory T cells in splenocytes following ATR inhibition, ICI therapy, and ablative radiotherapy

To determine whether ATR inhibition, ablative radiotherapy, and ICI therapy lead to protective T-cell immunity, CD44^+^ memory T cells among splenocytes were quantified (Fig. 6D). CD44^+^CD62L^–^ and CD44^+^CD62L^+^ cells were classified as effector and central memory T cells, respectively. The proportion of central and effector memory T cells increased in the treatment groups than that in the control group (*P* < 0.05), with high proportions noted in the irradiated group. These results support the hypothesis that ablative radiotherapy induces immunogenic cell death and immunological memory, and the addition of ATR inhibition and ICI further enhances protective T-cell immunity.

### Triple therapy was associated with minimal hepatotoxicity, nephrotoxicity, and hematological suppression

Mice treated with ablative radiotherapy or ATR inhibition with or without ICI therapy showed normal serum creatinine, blood urea nitrogen, alanine aminotransferase, and aspartate aminotransferase levels without nephrotoxicity or hepatotoxicity (Supplementary Fig. S1A). In terms of peripheral blood hematological parameters, mice subjected to ablative radiotherapy alone exhibited reduced levels of white blood cell count, hemoglobin, and hematocrit levels (Supplementary Fig. S1B), suggesting neutropenia and anemia. However, mice that received irradiation with ATR inhibition, with or without ICI therapy, showed normal levels of hematological parameters. Platelet counts did not differ among the groups. Collectively, triple therapy was safe, without significant hepatotoxicity, nephrotoxicity, or hematologic suppression. Individual monitoring of hematological parameters is, nevertheless, warranted.

## Discussion

Herein, we explored the effect of ATR inhibition on lung cancer in the context of ablative radiotherapy and immunotherapy. Combined ATR inhibition and ablative radiotherapy activated cGAS-STING-mediated immune signaling. Triple therapy induced immunogenic cell death and increased immune cell infiltration, with potential induction of protective T-cell immunity, highlighting the importance of combination therapies for improving outcomes.

Here, ATR inhibition elicited immunomodulatory effects. Ceralasertib (AZD6738) increased cytotoxic T-cell and NK cell infiltration while decreasing regulatory T-cell proliferation in the irradiated tumor microenvironment, promoting antitumor immunity in the lung epithelium (8 Gy in four fractions) and hepatocellular carcinoma (18 Gy in three fractions) [12, 23]. Berzosertib (VE-822) plus radiotherapy (5 Gy in a single fraction) enhanced type I interferon-related gene expression and immune activation to improve ICI efficacy in colorectal cancer [13]. These findings align with our results. Notably, the radiotherapy doses and fractionation schedules in these studies varied from conventional to hypofractionated but did not include an ablative schedule. This underscores the necessity for further investigation using an ablative radiotherapy schedule [4, 26]. Preclinical studies have demonstrated the superiority of ablative doses (≥ 10 Gy per fraction) in the induction of antitumor immune responses [27], with ablative radiotherapy transforming an immunosuppressive tumor microenvironment into an immunogenic microenvironment, characterized by cytotoxic T-cell infiltration and decreased immunosuppressive subpopulations. The selected ablative radiotherapy schedule in this study, combined with ATR inhibition and ICI therapy, yielded consistent and promising results.

In the present study, irradiation enhanced the expression of p-ATR in both human and mouse lung cancer cells, and ATR inhibition suppressed ATR phosphorylation levels in both non-irradiated and irradiated cells. While the total ATR levels varied among samples, the expression trends were consistent with those observed for p-ATR. These findings are consistent with those of the previous studies [18, 28], indicating that ATR levels could differ among various cell lines, and ATR was a critical regulator of the DNA damage response following irradiation. The ATR inhibitor may not only block ATR activity but also influence the expression of ATR protein itself, a hypothesis that warrants further investigation.

The sequencing of radiotherapy and immunotherapy has been investigated. Here, anti-PD-L1 treatment was initiated after the completion of radiotherapy. Radiotherapy enhances PD-L1 expression, and the completion of radiotherapy provides an optimal window for anti-PD-L1 therapy [4, 29]. Additionally, TILs might be adversely affected or depleted during ablative radiotherapy [30]. By administering anti-PD-L1 therapy after RT, the recruitment of new TILs post-radiotherapy could further enhance the immune response. The current sequencing strategy was designed to enhance the therapeutic efficacy of the combined treatment.

In our previously published report, the PD-L1 status in flank tumors was assessed using flow cytometry. High-dose ablative irradiation led to an increase in PD-L1 expression in tumor cells [4]. This finding underscores the significance of radiotherapy-induced enhancement of the PD-L1/PD-1 axis in the tumor microenvironment, which may contribute to tumor relapse and represents a key mechanism underlying acquired radioresistance. Modulating antitumor immunity can improve radiation responses by counteracting radiation-induced upregulation of immunosuppressive factors [4, 31]. ATR inhibition suppresses PD-L1 expression and mitigates PD-L1–PD-1 interactions, rendering cancer cells more susceptible to T-cell-mediated cytotoxicity [11, 14]. Furthermore, the phenotype of infiltrating immune cells might differ after ATRi treatment. ATRi treatment altered the activation status of tumor-infiltrating CD8 + T cells, resulting in downregulated expression of PD-1, LAG3, and Tim3, along with an increase in IFN-γ-secreting CD8 + T cells, thereby enhancing the cytotoxic activity of these cells [15, 23]. We will incorporate the exploration of infiltrating immune cell phenotypes in vivo in our future research.

The cGAS-STING pathway in tumor cells has been linked to the activation of the innate immune system, local activation of antigen-presenting cells, recruitment of effector lymphocytes, and sensitization of the tumor microenvironment to immune checkpoint inhibitor therapy [32]. In patients with NSCLC, upregulation of the cGAS-STING pathway has been associated with superior overall survival [33]. Consistently, we observed that simultaneous ATR inhibition and radiotherapy effectively activated the cGAS-STING pathway and overcame the immunosuppressive microenvironment, leading to enhanced antitumor immune responses when combined with ICI therapy.

The ability of nature killer subsets to kill tumor cells without prior antigen sensitization renders them attractive targets for anticancer immunotherapy. NK and NK T cells play central roles in maximizing treatment efficacy in the ablative radiotherapy-induced inflammatory tumor microenvironment [34]. Therefore, nature killer subsets can be used to identify patients with metastatic disease who may benefit from ICI therapy [35]. Our results revealed that combined ATR inhibition, ablative radiotherapy, and ICI increased natural killer cell infiltration in the irradiated flank tumor microenvironment, highlighting enhanced antitumor immune responses.

Here, combining ATR inhibition with radiotherapy was found to be safe, with no significant hepatotoxicity, nephrotoxicity, or hematologic suppression observed, consistent with existing preclinical and clinical data that suggest no increased normal tissue toxicity with this combination therapy [36, 37]. Notably, mice receiving ablative radiation alone experienced hematological suppression, whereas those receiving both ablative radiation and ATR inhibition did not. Given that ATR inhibition with berzosertib as monotherapy has demonstrated safety without inducing hematological suppression in human subjects [38], further investigation is needed to determine whether combining ATR inhibition with radiotherapy can mitigate radiation-induced myelosuppression.

In conclusion, ATR inhibition enhanced the efficacy of ablative radiotherapy and immunotherapy in lung cancer. Triple therapy induced immunogenic cell death, increased immune cell infiltration, and promoted protective T-cell immunity with minimal toxicity. Thus, leveraging the synergistic effects of ATR inhibition, ablative radiotherapy, and immunotherapy holds potential for enhancing patient survival and quality of life.

## Supplementary Information

Below is the link to the electronic supplementary material.

Supplementary file1 (DOCX 545 kb)

## Data Availability

The authors confirm that the data supporting the findings of this study are available within the article and its supplementary materials.
