# Supplementary material for: Combination of ataxia telangiectasia and Rad3-related inhibition with ablative radiotherapy remodels the tumor microenvironment and enhances immunotherapy response in lung cancer
Source: Cancer Immunol Immunother. 2024 Nov 2;74(1):8. doi: 10.1007/s00262-024-03864-6 (PMC11531452; doi:10.1007/s00262-024-03864-6)
Supplement: Supplementary file 1 — Supplementary file1 (DOCX 545 kb) [file 262_2024_3864_MOESM1_ESM.docx]

**
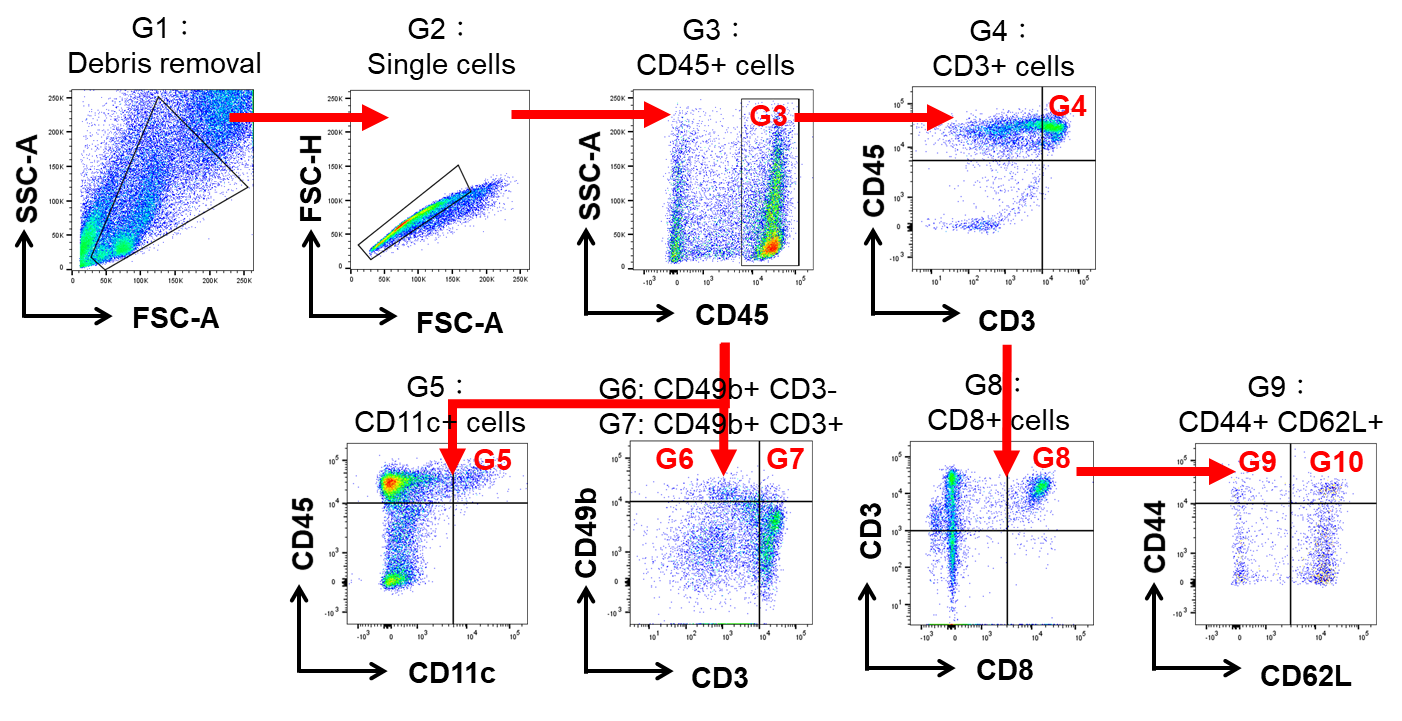
**

**Supplementary Figure S1.** **Gating strategies for flow cytometry analyses:** The flow cytometry gating strategy was initiated with CD45 pan-leukocyte gating (G1, G2, and G3), where leukocyte events were directed to the CD3 gate to quantify CD3+ T cells (G4). Leukocyte events from the CD45 pan-leukocyte gate were directed to the CD11c gate to quantify CD11c+ dendritic cells (G5). Additionally, within the CD45 pan-leukocyte gate, CD49b and CD3 gates were used to quantify CD3–CD49b+ natural killer cells (G6) and CD3+CD49b+ natural killer T cells (G7). Within the CD3+ T cell population, gating was further applied to distinguish CD4+ and CD8+ T cells, with the counting of CD8+ T cells (G8). Among CD8+ T cells, additional gating on CD44 and CD62L was performed to identify and quantify CD44+CD62L– effector T cells (G9) and CD44+CD62L+ central memory T cells (G10).


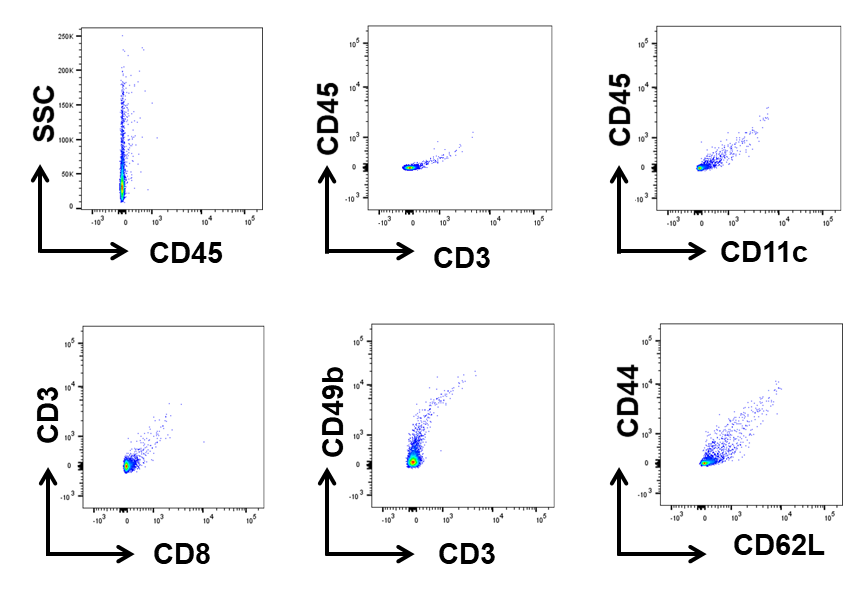


**Supplementary Figure S2.** Representative flow cytometric dot plot of unstained samples preparations used to define CD45 and SSC, CD3 and CD45, CD11c and CD45, CD8 and CD3, CD3 and CD49b, and CD62L and CD44 positive and negative populations.

**
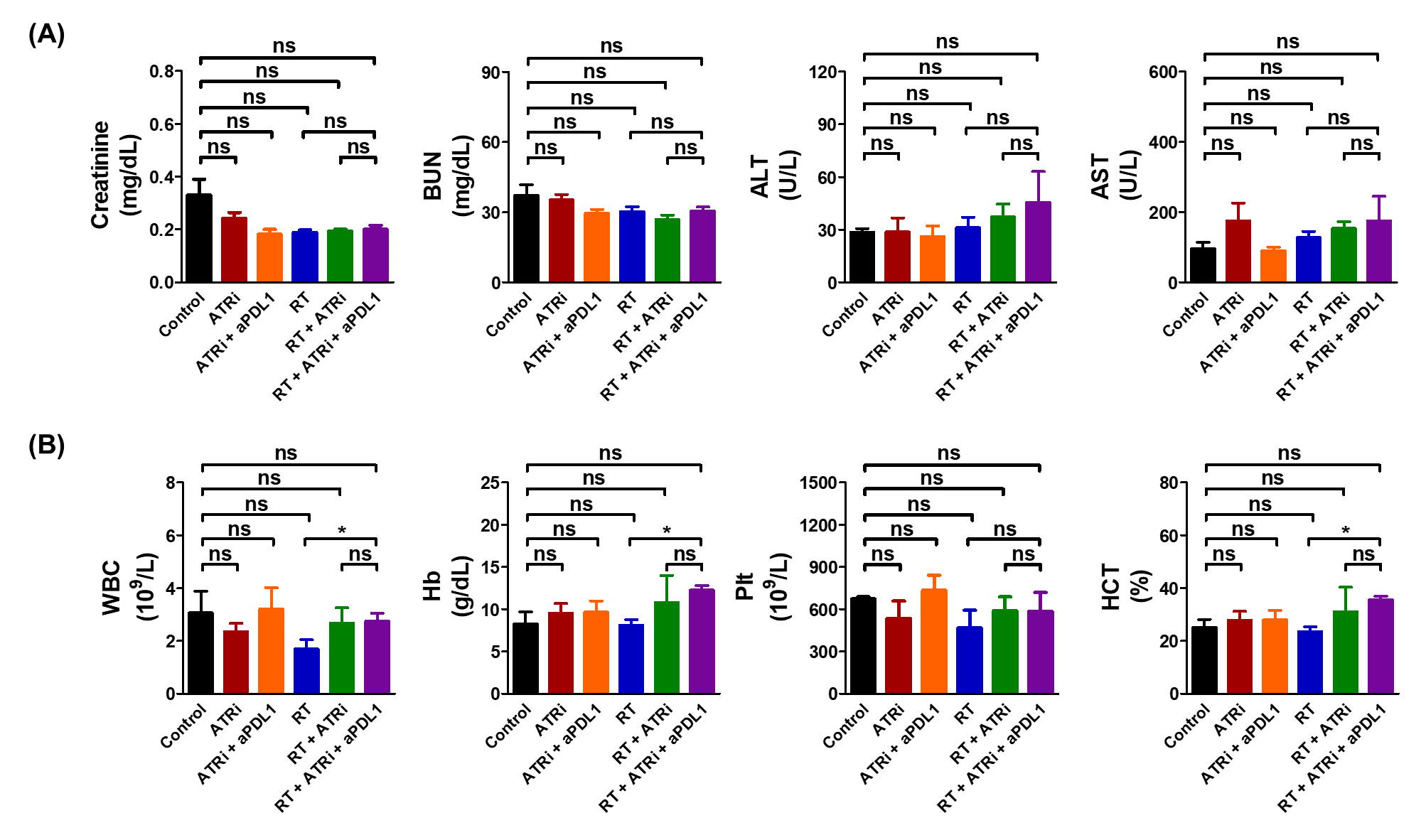
**

**Supplementary Figure S3.** Biochemical and hematological parameters in the peripheral blood of mice recorded eight days after ablative RT. (**A**) Creatinine, blood urea nitrogen (BUN), alanine aminotransferase (ALT), and aspartate aminotransferase (AST). (**B**) White blood cell count (WBC), hemoglobin (Hb), platelet count (plt), and hematocrit levels (Hct). Data are expressed as the mean ± standard error of the mean. **P* < 0.05; ns, not significant. The experimental groups comprised five mice per group.
